# Alpha-fetoprotein still is a valuable diagnostic and prognosis predicting biomarker in hepatitis B virus infection-related hepatocellular carcinoma

**DOI:** 10.18632/oncotarget.6913

**Published:** 2016-01-13

**Authors:** Mingjie Yao, Jingmin Zhao, Fengmin Lu

**Affiliations:** ^1^ State Key Laboratory of Natural and Biomimetic Drugs, Department of Microbiology and Infectious Disease Center, School of Basic Medicine, Peking University Health Science Center, Beijing, China; ^2^ Department of Pathology and Hepatology, Beijing 302 Hospital, Beijing, China

**Keywords:** alpha-fetoprotein (AFP), hepatitis B virus, hepatocellular carcinoma, diagnosis, Immunology and Microbiology Section, Immune response, Immunity

## Abstract

Use of serum alpha-fetoprotein (AFP) in clinical practices has been challenged in recent years, due to the lack of specificity and sensitivity. Here we conducted a retrospective study to evaluate the diagnostic and prognostic value of serum AFP among hepatocellular carcinoma (HCC) patients with their pathogenic features taken into consideration. The cohort for this study comprised 318 cases of hepatitis and 731 cases of cirrhosis, as well as 796 HCC patients. Using 11.62ng/mL as a cut-off value, the positive rate of AFP test among serum hepatitis B surface antigen (HBsAg) positive HCC patients was significantly higher than that in those HBsAg negative HCC patients (79.55% *vs* 56.49%, *P* < 0.000). Similarly, the median serum AFP level in HCC patients with serum HBsAg positive was significantly higher than that in those HBsAg negative HCC patients (423.89ng/ml *vs* 40.82ng/ml, *P* < 0.000). In addition, Kaplan-Meier curve analysis revealed that lower preoperative AFP level implicated a much higher overall survival rate. Of note, such prognosis predicting value was only seen in those chronic HBV infection-related HCC patients, but not among the HCC patients etiologically irrelevant to HBV infection. We believe that serum AFP is of diagnosis and prognostic predicting value for HCC with chronic HBV infection, and strongly suggest use of serum AFP as a biomarker in China and other HBV infection endemic area like Southeast Asia.

## INTRODUCTION

For decades, serum alpha-fetoprotein (AFP) is the most commonly used surveillance test for hepatocellular carcinoma (HCC) [1]. However, in recent years, use of serum AFP as a diagnosis and/or prognosis biomarker in HCC surveillance has been challenged in developed countries, due to the lack of specificity and sensitivity. Therefore, serum AFP measurement was no more recommended by European Association for the Study of the Liver (EASL) and American Association for the Study of Liver Diseases (AASLD) [2, 3]. In addition, some experts also questioned it as a valuable survival predicting biomarker.

It has been noticed that the incidence and etiology of HCC vary geographically and with different population. Recently, the etiological difference of serum AFP level between HBV and non-HBV infection-related HCC has been noticed by Chang Liu *et al* [4]. In addition, role of HBV x protein (HBx) on the up-regulation of alpha-fetoprotein receptor (AFPR) and AFP expression has been noticed in HBV-mediated liver cancer [5]. Our own data also suggested that viral co-transcription factor HBx could directly bind to and activate the promoter of AFP gene [6]. Based on these facts, it is reasonable to postulate that the serum AFP could still be a valuable biomarker in HBV infection-related HCC patients.

In this study, we conducted a retrospective study to evaluate the diagnostic and prognostic value of serum AFP measurement in Chinese HCC patients with different pathogenic features. Our results suggested that, despite all the pitfalls mentioned above, AFP is still a valuable biomarker for HCC diagnosis and for prognosis predicting in HBV infection-related HCC patients.

## RESULTS

**Figure 1:**
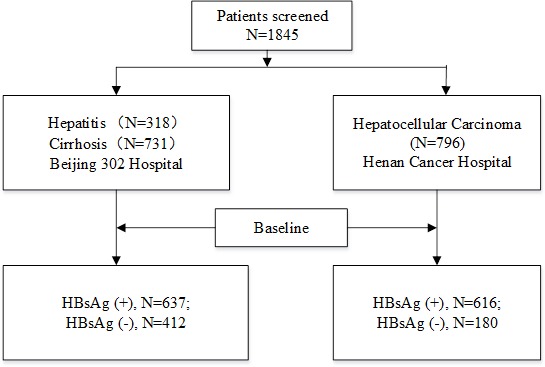
Patients’ flowchart, data provided in absolute numbers

A total of 1845 patients diagnosed either with chronic hepatitis, cirrhosis or HCC with different backgrounds were enrolled between December 2008 and December 2013 at Henan Cancer Hospital in Zhengzhou, and Beijing 302 Hospital (Figure 1). The cohort comprised 1467 (79.51%) males and 378 females (20.49%), including 318 cases of hepatitis (HBsAg+, *n* = 174; HBsAg-, *n* = 144), 731 cases of cirrhosis (HBsAg+, *n* = 463; HBsAg-, *n* = 268) and 796 HCC cases (HBsAg+, *n* = 616; HBsAg-, *n* = 180) (Table 1).

**Table 1:** Characteristics of patients in the present study

Factors		Hepatitis	Cirrhosis	HCC	*P*
**HBsAg(+)**					
**Gender**	**Male**	141 (81.03%)	373 (80.56%)	547(88.80%)	***0.000***
	**Female**	33(18.97%)	90(19.44%)	69(11.20%)	
**Age**	**Mean ± sd**	42.28±11.24	49.43±10.57	52.02±9.86	***0.000***
**HBsAg(−)**					
**Gender**	**Male**	81(56.25%)	197(73.51%)	128(71.11%)	***0.001***
	**Female**	63(43.75%)	71(26.49%)	52(28.89%)	
**Age**	**Mean ± sd**	47.13±11.77	53.60±10.49	57.02±11.09	***0.000***
**Total**	**N**	318	731	796	

**Figure 2:**
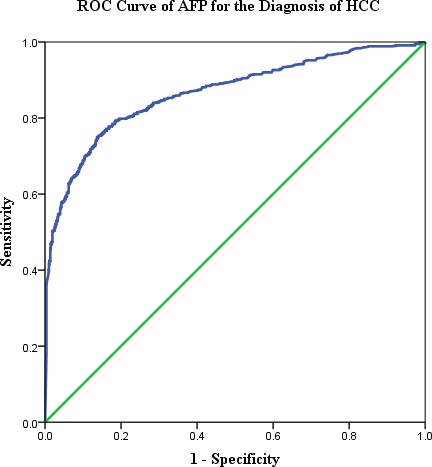
The receiver operating characteristic (ROC) curve of AFP in HCC diagnosis for all subjects The points in the ROC curve indicate different AFP values with corresponding sensitivity and specificity, from which 11.62ng/mL was chosen as the cut-off value for AFP.

Receiver operating characteristic (ROC) curves were plotted to identify a cut-off value that would best distinguish HCC patients from the other two groups of subjects. As showed in Figure 2, the optimal cut-off value for AFP was 11.62 ng/ml, which yielded a sensitivity of 74.94%, specificity of 86.29% and Youden index was 0.61, the area under the ROC curve was 0.866 (95% CI, 0.848-0.884, *P* < 0.000). Using 11.62ng/mL as the cut-off value of AFP level, the positive rate in HBsAg positive HCC patients was significantly than those HCC patients who were serum HBsAg negative (79.55% *vs* 56.49%, *P* < 0.000), when the etiological difference was taken into consideration. In line with this, with intervals set at >20ng/ml, >200ng/ml and >400ng/ml, the positive rates among those serum HBsAg positive HCC patients were 72.7%, 56.0% and 50.6%, respectively. Whereas, it dropped to 51.6%, 36.7% and 32.2% among those HCC patients who were serum HBsAg negative. The difference between the serum HBsAg posive and negative groups is of statistical significance (P < 0.000). And the median serum AFP level in HCC patients with serum HBsAg positive was significantly higher than that in those who were HBsAg negative (423.89ng/ml *vs*. 40.82ng/ml, *P* < 0.000). These results suggested that serum AFP levels were significantly elevated in a majority of HCC patients etiologically associated with chronic HBV infection.

**Figure 3:**
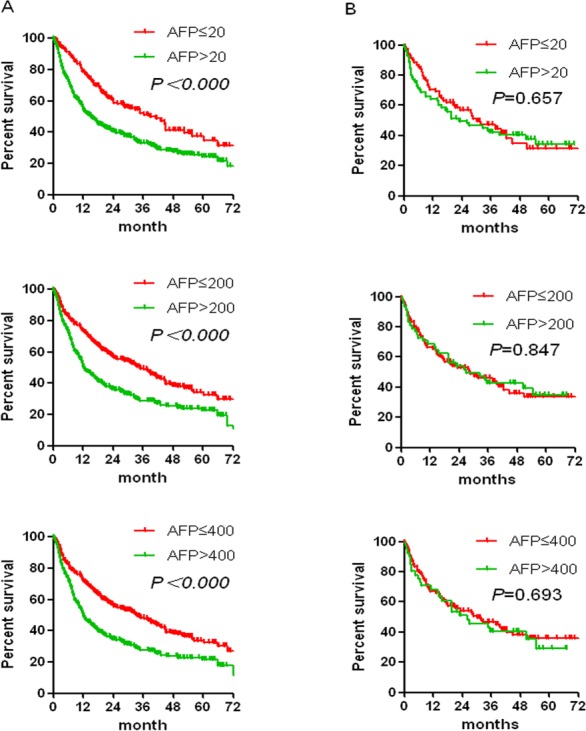
Cumulative post-surgery overall survival by different AFP levels among HCC patients with serum HBsAg positive (A) or with serum HBsAg negative (B) **A.** Cumulative incidence of HCC with HBV by different AFP levels. Comparison of cumulative survival among (0∼20 ng/ml and >20ng/ml, 0∼200 ng/ml and >200ng/ml, 0∼400 ng/ml and >400ng/ml) subgroups of patients. The cumulative survival probability of the patients at 1, 3, and 5 years were 65.49%, 42.96% and 30.51% (0∼20 ng/ml, *n* = 168), 40.35%, 25.69% and 13.78% (>20ng/ml, *n* = 448), 57.48%, 39.39% and 26.31% (0∼200 ng/ml, *n* = 271), 36.37%, 22.86% and 11.77% (>200 ng/ml, *n* = 345), 56.4%, 39.50% and 24.48% (0∼400 ng/ml, *n* = 304), 35.10%, 20.71% and 11.94% (>400 ng/ml, *n* = 312) respectively. **B.** Cumulative incidence of HCC with non-HBV by different AFP levels. Comparison of cumulative survival among (0∼20 ng/ml and >20ng/ml, 0∼200 ng/ml and >200ng/ml, 0∼400 ng/ml and >400ng/ml) subgroups of patients. The cumulative survival probability of the patients at 1, 3, and 5 years were 56.97%, 31.02% and 21.59% (0∼20 ng/ml, *n* = 77), 49.51%, 39.06% and 32.36% (>20ng/ml, *n* = 103), 52.81%, 32.47% and 25.06% (0∼200 ng/ml, *n* = 114), 53.71%, 40.11% and 31.20% (>200 ng/ml, *n* = 66),54.22%, 34.65% and 28.77% (0∼400 ng/ml, *n* = 122), 50.59%, 37.25% and 26.08% (>400 ng/ml, *n* = 58) respectively.

Additionally, the prognostic predicting value of AFP was evaluated through Kaplan-Meier method among a sub-cohort of 796 HCC patients who had underwent hepatectomy and had been followed up for a median of 34 months. As expected, lower preoperative AFP implicated a much higher overall survival rate among those HCC patients with evidence of chronic HBV infection (Figure 3). Of note, such prognostic predicating role of AFP was not seen in HCC patients without evidence of chronic HBV infection.

To investigate the risk factors for poor prognosis after surgical treatment further, univariate analysis and multivariate analysis (Cox regression) were performed with different pathogenic features taken into consideration. Univariate analysis showed that AFP value, tumor size, Gamma Glutamyl Transpeptidase (GGT) value, Alkaline phosphatase (ALP) value, and international normalized ratio (INR) value were independent risk factors affecting postoperative survival time among those HBsAg positive HCC patients. Interestingly, while among those HBsAg negative HCC patients, Barcelona Clinic Liver Cancer (BCLC) stage, INR value and Albumin (ALB) value, but not AFP value, were the independent risk factors affecting postoperative survival time. While the multivariate analysis revealed that AFP value, in together with INR value, GGT value, ALP value and tumor size, were the independent risk factors affecting overall post-surgery survival among those HBsAg positive HCC patients. While BCLC stage, INR value and ALB value were the independent risk factors for those HBsAg negative HCC patients (Table 2).

**Table 2:** Univariate and multivariate analysis for predictors of death

Risk Factor	HBsAg positive			HBsAg negative		
	HR	95%CI	*P* Value	HR	95%CI	*P* Value
**Univariate analysis**						
**Sex(Male/Female)**	1.008	0.757-1.342	0.955	1.236	0.765-1.998	0.387
**Age**	1.032	0.928-1.147	0.565	1.039	0.836-1.291	0.732
**No. of tumors**	1.227	1.117-1.349	***0.000***	1.274	1.052-1.542	0.013
**Diolame (Present/Absent)**	0.992	0.939-1.048	0.774	1.095	0.966-1.241	0.154
**Tumor size**	1.321	1.223-1.426	***0.000***	1.055	0.893-1.247	0.526
**BCLC**	1.196	1.068-1.340	***0.002***	1.568	1.227-2.004	0.000
**ALT**	1.009	0.982-1.036	0.519	1.048	0.834-1.316	0.688
**AST**	1.022	1.002-1.041	***0.029***	1.292	1.075-1.552	0.006
**ALP**	1.152	1.107-1.200	***0.000***	1.086	1.032-1.143	0.002
**GGT**	1.012	1.009-1.015	***0.000***	1.007	1.000-1.013	0.041
**AFP**	1.115	1.072-1.159	***0.000***	1.018	0.926-1.119	0.716
**INR**	1.932	1.258-2.966	***0.003***	3.164	1.673-5.984	0.000
**ALB**	0.375	0.228-0.617	***0.000***	0.178	0.040-0.805	0.025
**Multivariate analysis**						
**BCLC**				1.532	1.176 - 1.997	0.002
**AFP**	1.460	1.156 to 1.845	***0.002***			
**Tumor size**	1.240	1.103 to 1.394	***0.000***			
**GGT**	1.010	1.004 to 1.016	***0.001***			
**ALP**	1.102	1.017 to 1.195	***0.018***			
**INR**	1.806	1.140 to 2.861	***0.012***	3.491	1.874 - 6.052	0.000
**ALB**				0.186	0.040 - 0.864	0.032

Abbreviation: BCLC = Barcelona Clinic Liver Cancer, ALT = Alanine Transaminase, AST = Glutamic oxalacetic transaminase, ALP = Alkaline phosp, GGT = Gamma Glutamyl Transpeptidase, AFP = Alpha-fetoprotein, INR = International normalized ratio, ALB= Albumin.

## DISCUSSION

To date, the curative treatments of HCC include surgical resection, liver transplantation, TACE and RFA, etc. In order to improve patients’ prognosis and long-term survival, early diagnosis of HCC is essential to implement curative interventions [7]. Despite the disadvantage of low sensitivity, low specificity and limited accuracy in HCC early diagnosis, AFP has still been recommended as a serum biomarker for diagnosis of HCC in clinical practice in China, which accounts for more than 55% of annually diagnosed HCC patients.

A variety of factors have been reported affecting the prognosis of patients with HCC [3, 8, 9]. To provide evidence supporting the use of AFP measurement in clinical practices, particularly in China, here we evaluated the value of serum AFP measurement in clinical practices, with etiological differences of HCC development taken into consideration. Indeed, a majority (79.55%) of HCC patients with serum HBsAg positive had serum AFP levels greater than 11.62ng/ml, while which dropped to 56.49% in the HCC patients irrelevant to HBV infection. Additionally, among the HBV infection-related patients, the median serum AFP level in HCC patient group was significantly higher than that in CHB or cirrhosis patient (423.89ng/ml *vs* 40.82ng/ml, *P* < 0.000). Consistent with our observation, a report from Europe has indicated the different levels of serum AFP between HCC patients with and without HBV infection [10]. These results suggested that serum AFP levels are of diagnostic value for HCC patients with chronic HBV infection. This suggestion was supported by several recent studies which approved the predictive value of elevated serum AFP for the long-term risk of HCC development in chronic HBV infected patients [11, 12].

Serum biomarkers are attractive potential tools for HCC early diagnosis because they will enable non-invasive, objective and reproducible assessments. Considering the differences of tumorigenesis and clinical features between HBV-related HCC and non-HBV-related HCC, it is worthwhile to evaluate the prognosis predicting value of AFP in patients with HBV-related HCC, especially in Asian countries where HBV infection is endemic and a main causative factor of HCC. Indeed, in the current study, through univariate and multivariate analysis, we identified that preoperative AFP level was an independent risk factor for poor prognosis in patients with HBV-related HCC after surgical treatment, and lower preoperative AFP value implicated a much higher overall survival rate. Of note, such prognostic predicating value was only seen among those HCC patients with evidence of chronic HBV infection, but not among the HCC patients etiologically irrelevant to HBV infection.

In conclusion, we believe that serum AFP levels are of diagnosis and prognostic value for HCC patients with chronic HBV infection. We acknowledge that the present study is of limitations and need to be confirmed in a larger sample sized, controlled, multicenter and prospective study.

## MATERIALS AND METHODS

At enrollment, patient characteristics, virological, hematological, biochemical, and histological data were collected. Patients were examined for HCC by abdominal ultrasonography, dynamic CT, and/or MRI every 3-6 months. To evaluate the diagnostic and prognostic value of AFP, in this retrospective study, last time examination data of serum AFP level of non-HCC patients and data before therapy for HCC patents were collected. The measurement of AFP in the two hospitals were achieved by using same electrochemiluminescence immunoassay system Modular E170 (Roche, Mannheim, Germany), the normal range is 0 to 20 ng/ml. Decisions regarding to each patient's course of treatment were made based on the treatment guidelines for HCC in China [13]. The diagnosis of HCC was confirmed by pathologic examination of the resected liver specimens. HBV infection status was based on serum hepatitis B surface antigen (HBsAg). Diagnosis of cirrhosis was based on liver histology or clinical, laboratory and imaging data. All 796 HCC patients had underwent hepatectomy and were followed up for a median of 34 months. The prognostic predicting value of AFP was evaluated through Kaplan-Meier method and differences among different groups were assessed using the log-rank test.

All statistical analyses were performed using the statistical software package SPSS version 21.0 for Windows (SPSS, Chicago, Illinois, USA). All tests of significance were two-tailed and *P* < 0.05 was considered statistically significant.

The study protocol was approved by the institute ethics committee, and the informed consents were obtained from all patients and donors before the start of study.
